# Mixed contaminant exposure in tapwater and the potential implications for human-health in disadvantaged communities in California

**DOI:** 10.1016/j.watres.2024.122485

**Published:** 2024-09-25

**Authors:** Kelly L. Smalling, Kristin M. Romanok, Paul M. Bradley, Michelle L. Hladik, James L. Gray, Leslie K. Kanagy, R. Blaine McCleskey, Diana A. Stavreva, Annika K. Alexander-Ozinskas, Jesus Alonso, Wendy Avila, Sara E. Breitmeyer, Roberto Bustillo, Stephanie E. Gordon, Gordon L. Hager, Rena R. Jones, Dana W. Kolpin, Seth Newton, Peggy Reynolds, John Sloop, Andria Ventura, Julie Von Behren, Mary H. Ward, Gina M. Solomon

**Affiliations:** a U.S. Geological Survey, Lawrenceville, NJ, USA; b U.S. Geological Survey, Columbia, SC, USA; c U.S. Geological Survey, Sacramento, CA, USA; d U.S. Geological Survey, Lakewood, CO, USA; e U.S. Geological Survey, Boulder, CO, USA; f National Cancer Institute, National Institutes of Health, Rockville, MD, USA; g Sierra Streams Institute, Nevada City, CA, USA; h Clean Water Action/Clean Water Fund, Oakland, CA, USA; i Communities for a Better Environment, Los Angeles, CA, USA; j U.S. Geological Survey, Downingtown, PA, USA; k U.S. Geological Survey, Kearneysville, WV, USA; l U.S. Geological Survey, Iowa City, IA, USA; m Office of Research and Development, U.S. Environmental Protection Agency, Research Triangle Park, NC, USA; n University of California San Francisco, San Francisco, CA, USA; o ORISE, Office of Research & Development, U.S. Environmental Protection Agency, Research Triangle Park, NC, USA; 1 Current Affiliation: National Institute of Environmental Health Sciences.

**Keywords:** Drinking water, Tapwater, Contaminant mixtures, Disadvantaged communities, Health-effects, California

## Abstract

Water is an increasingly precious resource in California as years of drought, climate change, pollution, as well as an expanding population have all stressed the state’s drinking water supplies. Currently, there are increasing concerns about whether regulated and unregulated contaminants in drinking water are linked to a variety of human-health outcomes particularly in socially disadvantaged communities with a history of health risks. To begin to address this data gap by broadly assessing contaminant mixture exposures, the current study was designed to collect tapwater samples from communities in Gold Country, the San Francisco Bay Area, two regions of the Central Valley (Merced/Fresno and Kern counties), and southeast Los Angeles for 251 organic chemicals and 32 inorganic constituents. Sampling prioritized low-income areas with suspected water quality challenges and elevated breast cancer rates. Results indicated that mixtures of regulated and unregulated contaminants were observed frequently in tapwater throughout the areas studied and the types and concentrations of detected contaminants varied by region, drinking-water source, and size of the public water system. Multiple exceedances of enforceable maximum contaminant level(s) (MCL), non-enforceable MCL goal(s) (MCLG), and other health advisories combined with frequent exceedances of benchmark-based hazard indices were also observed in samples collected in all five of the study regions. Given the current focus on improving water quality in socially disadvantaged communities, our study highlights the importance of assessing mixed-contaminant exposures in drinking water at the point of consumption to adequately address human-health concerns (e.g., breast cancer risk). Data from this pilot study provide a foundation for future studies across a greater number of communities in California to assess potential linkages between breast cancer rates and tapwater contaminants.

## 1. Introduction

California (United States, U.S.) residents rely on drinking water from both surface-water and groundwater sources, with relative sourcing depending on the region of the state, water needs, resource availability, and climate. Of the 39 million people in California, 95 % receive their water from Federally-regulated public water supplies (PWS), with the remainder (~ 2 million people) relying on generally unregulated private wells or small water systems (Bangia et al., 2020; Pace et al., 2022; State Water Resources Control Board, 2021). PWS are defined as systems that have either 15 or more service connections or systems that serve at least 25 people daily for at least 60 days out of the year (State Water Resources Control Board, 2021). Quality, sustainability, regulation, and compliance monitoring are fundamental public-health challenges for California’s drinking-water infrastructure, which includes approximately 7000 PWS with service populations ranging from fewer than 600 up to >1 million people (Reibel et al., 2021). Only about seven percent of the PWS in California serve communities with >10,000 people while the majority (~83 %) of the PWS serve communities with <1000 people (State Water Resources Control Board, 2021).

In 2012, California became the first state to pass a Human Right to Water Law which recognized the right of every resident to clean, safe, and affordable drinking water, whether from public- or private-supply (State Water Resources Control Board, 2021). There are many challenges in achieving this basic right, including the effective removal of various contaminants, the costs incurred in treating drinking water, and the general dependence on unregulated private wells in rural areas. Similar to other regions across the U.S., mixtures of regulated (e.g., arsenic, nitrate, disinfection byproduct(s) (DBP), volatile organic compound(s) (VOC), five per- and polyfluoroalkyl substances (PFAS)) and unregulated contaminants such as pharmaceuticals, many PFAS, and some pesticides (Balazs et al., 2011, 2012; Burow et al., 2008; Fram and Belitz, 2011) are present in drinking water supplies in many areas of California. Arsenic and uranium are naturally occurring and are among the most common contaminants observed in California, particularly in groundwater from the Central Valley (Balazs et al., 2012; Lopez et al., 2021; Rosen et al., 2019). In the Central Valley and other agricultural regions of the state, the application and leaching of surface-applied fertilizers, animal waste, and pesticides are also sources of nitrate and pesticide contamination to local aquifers and surface water systems (Burow et al., 2008; London et al., 2021). Lastly, industrial activities and wastewater discharges, particularly in urban areas, can be sources of a wide range of contaminants (e.g., VOC, PFAS, and pharmaceuticals) to drinking-water resources (Fram and Belitz, 2011; Hu et al., 2016).

Documented human exposures to complex mixtures of contaminants in drinking water and concomitant potential for chronic human-health effects even at low levels (Bradley et al., 2020, 2018, 2023; Bradley et al., 2021a, 2021b, 2022; Smalling et al., 2023a, 2023b) demonstrate the importance of continued monitoring of drinking water at the point of consumption (tapwater) for both regulated and unregulated contaminants, particularly in disadvantaged communities disproportionately affected by poor water quality (Allaire and Acquah, 2022; Allaire et al., 2018; Meltzer et al., 2020; Mueller et al., 2024; Patel and Schmidt, 2017; Schaider et al., 2019; VanDerslice, 2011; Zheng and Flanagan, 2017). For example, large PWS serving >10,000 people in low income communities were more likely to have higher concentrations of chemicals like arsenic and nitrate in their finished water compared to systems serving higher income communities (Balazs et al., 2011, 2012). Further, small water systems in rural areas are more likely to see detections of multiple contaminants, tend to have more maximum contaminant level (MCL) violations and are financially strained due to lower incomes and declining populations compared to their larger counterparts (Allaire and Acquah, 2022; Bangia et al., 2020). These systems are also less likely to support advanced treatment, have less technical capacity and limited financial resources to support/implement infrastructure upgrades (Allaire and Acquah, 2022).

In California, over 2000 National Primary Drinking Water Regulation violations were reported by PWS in 2021, including 753 MCL violations (State Water Resources Control Board, 2022). Inorganic contaminants (e.g., nitrate, arsenic) were responsible for the largest number of violations, followed by DBP and radionuclides (State Water Resources Control Board, 2022). Approximately, 92 % of these violations occurred in smaller PWS that serve rural low-income communities with fewer than 500 service connections, non-transient non-community water systems (e.g., schools), or transient non-community water systems (e.g., campgrounds). In 2018 over 1 million people were estimated to lack access to safe drinking water (e.g., meeting all state and federal drinking water standards) in California (Pannu, 2018) including those on regulated PWS and unregulated private wells or water systems with <15 service connections.

Breast cancer is one of the most common cancers among women worldwide and in the U.S., accounting for about 25 % of all cancer cases in women (Heer et al., 2020; Wilkinson and Gathani, 2022). A number of epidemiological studies have examined links between breast cancer risk and certain legacy environmental contaminants in (e.g., organochlorine pesticides, and polychlorinated biphenyls; Negri et al., 2003; Snedeker, 2001). More recently, many chemicals with estrogen-modulating effects were also found to be carcinogenic or mutagenic (Choi et al., 2004; Yoon et al., 2014). Chemical contaminants found in U.S. drinking-water supplies (e.g., PFAS, triclosan, phthalates, etc.) have been shown to cause mammary tumors in animals (Siddique et al., 2016) but few epidemiologic studies have evaluated exposure to specific drinking-water contaminants and breast cancer (Aschengrau et al., 2003; Gallagher et al., 2010; Rodgers et al., 2018). However, only a fraction of potential carcinogens is routinely monitored in drinking water. As a first step toward understanding the potential risk of breast cancer from exposure to drinking-water contaminants, there is a benefit to screening drinking water for both regulated contaminants as well as unmonitored and unregulated chemicals that are known or suspected carcinogens, mammary toxicants, or endocrine disruptors (Kay et al., 2024).

Building upon information on community socioeconomic status (United States Census Bureau, 2023), drinking-water contaminant information from the California EPA [CalEnviroScreen 3.0 https://oehha.ca.gov/calenviroscreen/indicator/drinking-water-contaminants], and breast cancer patterns from the California Cancer Registry (CCR, ccrcal. org), this initial pilot study was designed to assess the prevalence of drinking water contaminant mixtures in tapwater samples collected from disadvantaged communities in several regions of California. In our paper we define disadvantaged communities as those with median household incomes less than $25,000, with suspected drinking water quality challenges, or elevated breast cancer rates. The study was also designed to screen tapwater for potential endocrine activity utilizing several in vitro bioactivity-based approaches.

## 2. Materials and methods

### 2.1. Sample collection information

**Fig. 1.**
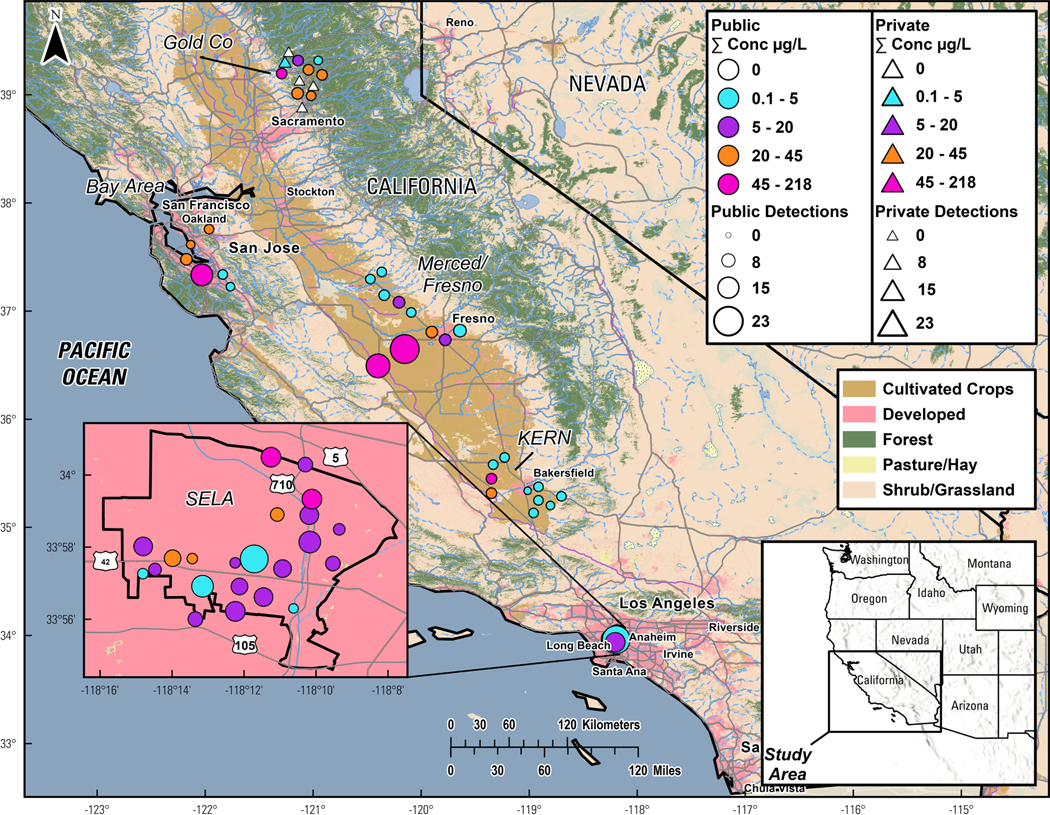
Cumulative (sum of all detected) concentrations (μg/L) and numbers of organic compounds detected in private well (*n* = 5) and public supply (*n* = 55) samples collected in 2020–21 from five regions in California. For site details see Table S1 and organic results see Table S3. National Landcover data is available at https://doi.org/10.5066/P9KZCM54 (Dewitz and U.S. Geological Survey, 2021). Base map image is from ESRI (ESRI Data & Basemaps, 2023) and Southeast Los Angeles Boundary (inset) is from the South Coast Air Quality Management District’s (SCAQMD) open data portal at https://data-scaqmd-online.opendata.arcgis.com/ (SCAQMD, 2023).

A total of 60 tapwater samples from private residences in rural and urban communities were collected from five geographic regions in California (Fig. 1, Table S1) in 2020–2021 (Romanok et al., 2021). The geographic regions included Gold Country (*n* = 12), the San Francisco Bay Area (*n* = 6), the Central Valley including Merced/Fresno (*n* = 10) and Kern (*n* = 10) counties and southeast Los Angeles, hereafter SELA (*n* = 22; Fig. 1, Table S1).

To select specific PWS and private wells for sampling within these five regions, we used PWS boundary maps supplemented by groundwater data at the township level. Specific geographic areas of focus for participant recruitment were selected based on the intersection of three main criteria including 1) prior detection of indicator contaminants (California Office of Environmental Health Hazard Assessment, 2020), 2) breast cancer rates (California Cancer Registry, 2018), and 3) neighborhood socioeconomic status (United States Census Bureau, 2023). We obtained data from the State of California for 21 indicator contaminants in California water systems over a nine-year period (as specified below). The presence of any of these contaminants during that time indicated a greater likelihood of compromised water quality, and the potential presence of other unmeasured contaminants. Evaluation of prior contaminant detections included any reported detections within the past 9 years (2010–2019) of hexavalent chromium, cadmium, lead, dibromochloropropane, perchlorate, several VOC (perchloroethylene, trichloroethylene, trichloropropane), any of 9 reported PFAS; or any reported concentration more than half the MCL for nitrate, arsenic, uranium or radium (California Office of Environmental Health Hazard Assessment, 2020). Breast cancer is the most common cancer among women and the second leading cause of cancer deaths among women in California with over 30,000 new cases diagnosed yearly (California Cancer Registry, 2018). In 2012 the California Breast Cancer Mapper Project (CBCMP) produced a series of maps identifying areas, irrespective of county boundaries, where the incidence of invasive breast cancer is 10–20 % higher than the rest of the state (Tracking California, 2012). For the breast cancer criteria, we used census tracts identified by the CBCMP where the age-adjusted incidence was at least 10 % higher than the rest of the state between 2000 and 2008 (Roberts et al., 2013; Tracking California, 2020). Finally, low income communities were identified by census tracts where greater than 20 % of the population had household incomes less than $25,000 utilizing American Community Survey 5-Year Data from 2017 (United States Census Bureau, 2023). A detailed individual socioeconomic and health survey was not conducted with participating households, primarily because sample locations were selected to inform the water quality within a respective community’s water supply, consistent with the designed drinking-water exposure focus of the study, but also because of limited sample numbers (high per sample analytical costs, extant Covid-19 pandemic condition) and corresponding participatory-bias concerns (Gibson and Pieper 2017; Wait et al. 2020). For this study, we consider the individual residences selected as representative of the community’s water supply.

Communities where census tracts with elevated breast cancer rates and low-income neighborhoods intersected with potentially contaminated PWS or township boundaries (for private wells in rural areas where there were PWS) were prioritized for potential sampling. We sampled water from PWS or private wells serving these types of areas, rather than having a selection strategy based on individuals. All communities selected in the study met a minimum of two of the three criteria (see below for details) and were defined as disadvantaged for the purposes of this paper. Specifically, the selected census tracts in each region, met the water quality concern criterion, plus either the income or the breast cancer rate criterion. In practice, the census tracts selected in Gold Country and SELA met all three study criteria, but the selected census tracts in the Central Valley are mostly in low breast cancer rate areas, so those only met two criteria. The census tracts selected in the Bay Area met all three criteria with only one exception.

Gold Country (northeastern California in the Sierra Nevada foothills) is predominantly rural with potential drinking-water concerns from past mining activities and recent wildfires. The area is characterized by small PWS (population served: 330–47,200; Table S1) and private wells, a high incidence of breast cancer, and low-income communities. San Francisco Bay area samples were collected from communities in the southern portion of the region (Fig. 1), which has some of the highest breast cancer rates in the state. California’s Central Valley is predominantly agricultural with a high quantity of pesticide use, a reliance on groundwater as a drinking-water source, and many low-income communities. Sampling in the Central Valley region was conducted in three counties including Merced and Fresno counties in the northern portion and Kern County in the southern portion (Fig. 1; Table S1). Lastly, we collected samples from SELA, a highly urbanized/industrialized area with low-income communities and above-average rates of breast cancer. Regions were selected for sampling, in part, due to the presence of community-based partner organizations engaged in all phases of the research project (e.g., planning, participant recruitment, sample collection, and results-return) in each region: Sierra Streams Institute in Gold Country, Clean Water Fund in Kern County, and Communities for a Better Environment in SELA; staff from the Public Health Institute conducted recruitment and sampling in the northern Central Valley and the San Francisco Bay Area.

Within each area, households were recruited to participate using a variety of methods, including flyers, recruitment at central community locations, email, and social media outreach, going door to door, and word-of-mouth. When individuals expressed interest, their address was checked against the map to ensure their location fell within the selected census tracts prior to enrollment. Tapwater samples were collected from 55 residences receiving their water from PWS and 5 residences on private wells (all in Gold Country; Table S1). Drinking-water sources for PWS varied by geography and location (Table S1). Ten homes relied on drinking water sourced from surface waters, 18 relied on groundwater, while 27 locations relied on mixed sources (Table S1). PWS service areas ranged from 367 (Central Valley) to 3.95 million (SELA) people (Table S1) and comprised 19 small (<10,000 people served) and 36 large (≥10,000 people served) systems.

Due to Covid-19 restrictions, sampling kits and instructions were prepared and shipped to community partners who visited and remained outside each residential sampling location, guided homeowner collection of kitchen-faucet tapwater samples, and completed sample processing, packaging, and shipment of samples to the laboratory. Samples were collected one time between November 2020 and May 2021 with sample times varying throughout the day and without precleaning, screen removal or flushing of the sample tap and not comparable with the lead/copper rule sampling for compliance monitoring (U.S. Environmental Protection Agency, 2008). During sampling each participant was also asked to complete a brief survey on their use of unfiltered tapwater. Participants were asked if they used unfiltered tapwater for a) drinking, b) cooking/making hot beverages, c) only other activities (e.g., laundry, washing dishes, pets, gardening) or d) not at all. The study was conducted in accordance with the Declaration of Helsinki and approved by the Institutional Review Board of the Public Health Institute, IRB #I19–001, January 6, 2019. The study participants provided written informed consent.

### 2.2. Analytical methods and quality assurance

Tapwater samples were analyzed by the USGS for 251 unique organic compounds using three targeted methods (Furlong et al., 2014; Hladik et al., 2014; Kolpin et al., 2021; Sandstrom et al., 2015) and 32 inorganic constituents using three targeted methods; (Brinton et al., 1995; Hergenreder, 2011; McCleskey et al., 2003; U.S. Environmental Protection Agency, 2014) as discussed in detail previously (Bradley et al., 2020, 2018, 2021a; Romanok et al., 2018). Organic analytes included DBP, pesticides, PFAS, and pharmaceuticals; additional method details are in the Supporting Information (Table S2). Bottles for pharmaceutical analysis were pretreated with ascorbic acid to neutralize chlorine/chloramine. Detailed information on analytes and detection limits for each of the methods are available in (Romanok et al., 2021) and Table S2.

All tapwater samples were also analyzed for in vivo androgen (AR), glucocorticoid (GR), thyroid (TR), aryl hydrocarbon (AhR) and estrogen (ER) receptor bioactivities (see Figures S3-S7) by the National Cancer Institute (NCI) using mammalian-cell phenotypic bioassays based on quantitative imaging of translocation of green-fluorescent-protein (GFP) labeled nuclear-receptor chimeric constructs from the cytoplasm to the nucleus (Jones et al., 2020; Stavreva et al., 2021).

Quantitative (≥ limit of quantitation, ≥ LOQ) and semi-quantitative (between LOQ and long-term method detection limit, MDL) results were treated as detections (Childress et al., 1999; Foreman et al., 2021; Mueller et al., 2015). Quality-assurance/quality-control included analyses of 10 field blanks including two blanks in each region except for the Bay Area where only one was collected, laboratory spikes, and stable isotope surrogates. Fifteen inorganic constituents and two organic compounds were detected in field blanks (Table S5). All tapwater samples collected from a region were censored to the highest values detected in the blank(s) collected from that region. Across all regions, field blank detections resulted in censoring for nitrate (in 11 samples), copper (in 5 samples), iron (in 7 samples), manganese (in 2 samples) and zinc (in 12 samples) (Table S5). Only two organic compounds (metolachlor, tiotropium) were (observed once each) in field blanks; tiotropium was detected in a single tapwater sample at the corresponding field-blank concentration and removed from the interpretive dataset (Table S5). The median surrogate recovery for organic analytes was 101 % (interquartile range 89.9–110 %; Table S6).

### 2.3. Individual contaminant comparison to federal drinking water regulations

Because California’s drinking water regulations for the contaminants observed herein were similar to the EPA’s National Primary Drinking Water Regulations, we used the EPA MCL for regulatory context only and as a frame of reference for private well tapwater (Table 1; Tables S3 and S4). MCL are only enforceable in public supply and compliance monitoring is often conducted at the PWS prior to distribution to the service area (U.S. Environmental Protection Agency, 2018; U.S. Environmental Protection Agency, 2023). Because, MCL values take into account both technical and financial limitations associated with drinking-water treatment (U.S. Environmental Protection Agency, 2024a), the potential for apical human-health effects of individual contaminant exposures was screened based on the MCL goal(s) (MCLG), “the maximum level of a contaminant in drinking water at which no known or anticipated adverse effect on the health of persons would occur, allowing an adequate margin of safety,” when considering sensitive (infants, children, elderly, immune- or disease-compromised) sub-populations (U.S. Environmental Protection Agency, 2024a), and other similar state and international drinking-water human-health advisories.

### 2.4. Cumulative contaminant effects-based screening approaches

A screening-level assessment (Goumenou and Tsatsakis, 2019; U.S. Environmental Protection Agency, 2011a) of potential cumulative biological activity of chemical mixtures in each tapwater sample was conducted using two analogous bioactivity-weighted approaches as described previously (Blackwell et al., 2017; Bradley et al., 2019, 2018). These approaches follow well documented and common risk screening/assessment approaches (More et al., 2019; Price, 2023; Price et al., 2020) designed to provide relevant insight into the cumulative risk of the large numbers of chemical components in environmentally and human health relevant mixture exposures. The exposure-activity ratio (EAR) approach is a considered a high-level screening of the potential for molecular-scale vertebrate effects of organic compounds and is considered a complement the Hazard Index (i.e., toxicity quotient; TQ) approach for both organic and inorganic contaminants. The ToxCast vertebrate-centric in vitro effects library was specifically assembled to inform estimates of human exposure-response relations at the site of molecular activity (Blackwell et al., 2017). The EAR approach has been employed previously in both drinking water (Bradley et al., 2020, 2018) and surface waters (Blackwell et al., 2018; Corsi et al., 2019) and serves as a reasonable first-level estimate of in vivo molecular-level effects potential but does not directly translate to apical human-health endpoints. The ToxEval version 1.3.0 (De Cicco et al., 2018) was used to sum (non-interactive, concentration addition model, e.g. Altenburger et al., 2018; Cedergreen et al., 2008; Stalter et al., 2020) individual EAR from the Toxicity ForeCaster (ToxCast, high-throughput screening data; U.S. Environmental Protection Agency Center for Computational Toxicology and Exposure, 2022) to estimate sample-specific cumulative EAR (∑EAR; Blackwell et al., 2017; Bradley et al., 2018). EAR is the ratio of the detected concentration in the sample to the activity concentration at cutoff (ACC) obtained from the ToxCast database. The ACC estimates the point of departure concentration at which a defined threshold of response (cutoff) is achieved for a given biological activity and is less prone to violations of relative potency assumptions (Blackwell et al., 2017). ACC data in the ToxEval v1.3.0 employed in the present study were from the August 2022 invitroDBv3.5 release of the ToxCast database (U.S. Environmental Protection Agency Center for Computational Toxicology and Exposure, 2022). Non-specific endpoint, baseline, and unreliable response-curve assays were excluded (Blackwell et al., 2017; Bradley et al., 2018). A ∑EAR=1 indicates a level that is expected to modulate a molecular target in vitro while a ∑EAR=0.001 is considered a precautionary screening level of interest. ∑EAR results and exclusions are summarized in Tables S7-S9.

Because the ∑EAR approach was limited to organic compounds, an analogous human-health-based Hazard Index assessment (Goumenou and Tsatsakis, 2019; U.S. Environmental Protection Agency, 2011a; 2012) of cumulative organic and inorganic contaminant risk was also conducted to sum (non-interactive concentration addition model Altenburger et al., 2018; Cedergreen et al., 2008; Stalter et al., 2020) the TQ (ratio of detected concentration to corresponding health based benchmark) of individual detections to estimate sample-specific cumulative TQ (∑TQ; Corsi et al., 2019). A precautionary screening-level approach was employed based on the most protective human-health benchmark (i.e., lowest benchmark concentration) among MCLG (U.S. Environmental Protection Agency, 2023), World Health Organization (WHO) guideline values (GV) and provisional GV (pGV) (World Health Organization (WHO), 2011), USGS health-based screening level (HBSL; Norman et al., 2018), and other available state benchmarks (Table S10). For the ∑TQ assessment, MCLG values of zero (i.e., no identified safe-exposure level for sensitive sub-populations, including infants, children, the elderly, and those with compromised immune systems and chronic diseases; U.S. Environmental Protection Agency, 2024a; U.S. Environmental Protection Agency, 2023) were set to 0.1 μg/L for any DBP, arsenic, lead, uranium and 0.0001 μg/L for PFOA and PFOS. Due to the inclusion of a margin of safety in health benchmarks, a ∑TQ=1 indicates a high probability of risk while a ΣTQ<0.1 indicates no risk. ∑TQ results and respective health-based benchmarks are summarized in Tables S10-S11. Screening assessments were conducted in the program R version 4.3.1 (R Development Core Team, 2019).

### 2.5. Statistical analysis

Differences (centroids and dispersions) in contaminant concentrations among regions, PWS size (small and large) and source water types (surface water, groundwater, and mixed) were assessed by one-way PERMANOVA (*n* = 9999 permutations) on Euclidean distance (Hammer et al., 2001). When differences were detected by PERMANOVA, a Kruskal–Wallis one-way analysis of variance by ranks with a Dunn pairwise post hoc test with Bonferroni correction on medians was performed to determine which pairs were significantly different from each other (Piepho, 2004). For statistical analysis only, all non-detections were assigned a value below the lowest detected value as follows. For pesticides, pharmaceuticals and PFAS the non-detections were assigned a value of 0.0001 μg/L; cumulative organics and DBP non-detections were assigned a value of 0.01 μg/L. For manganese, nitrate, and lead, non-detects were set to 0.01 μg/L and were set to 0.1 ug/L for arsenic and uranium.

## 3. Results

**Fig. 2.**
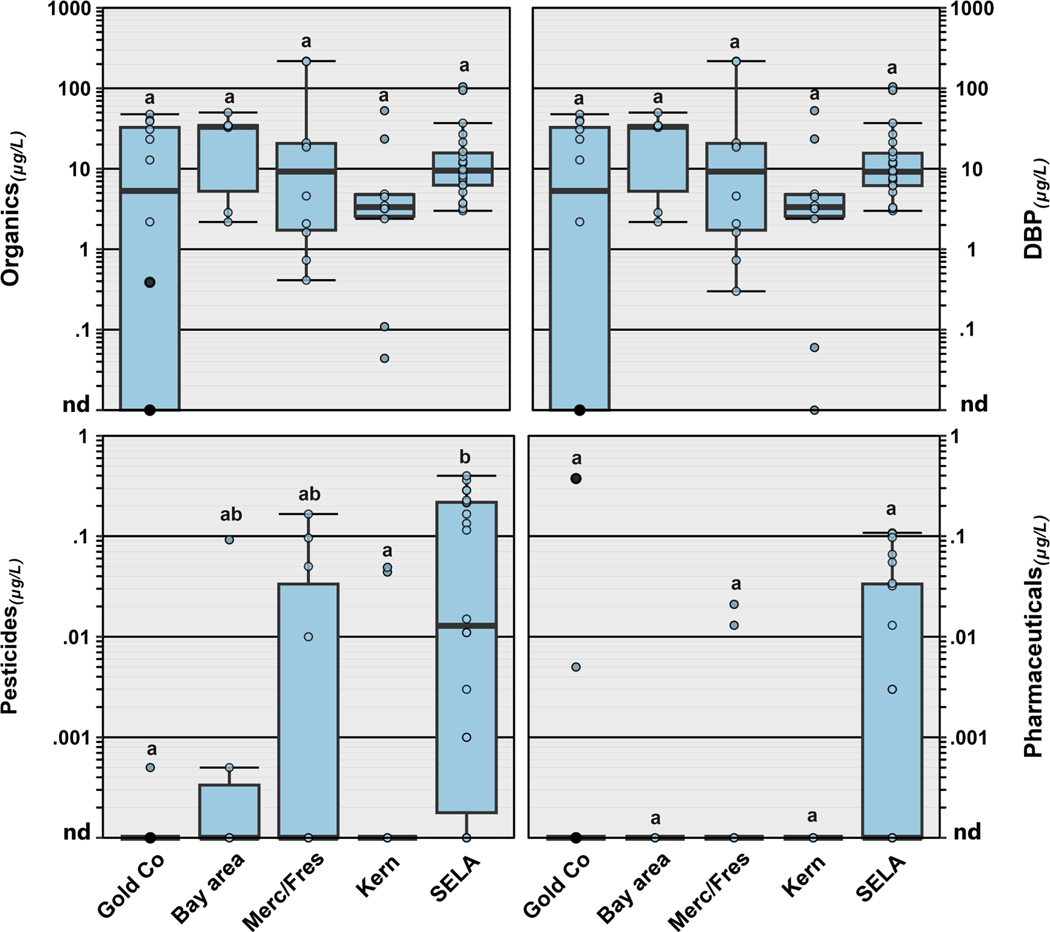
Concentrations (μg/L) of cumulative organics (top left), disinfection byproducts (DBP; top right), pesticides (bottom left), and pharmaceuticals (bottom right) in tapwater samples collected in 2020–21 from five regions of California including Gold Country (Gold Co), the San Francisco Bay Area (Bay area), Merced and Fresno counties (Merc/Fres), Kern County (Kern) and southeast Los Angeles (SELA). Private well samples collected from Gold Country are represented by closed circles. Open circles are data for individual public supply samples. Boxes, centerlines, and whiskers indicate interquartile range, median, and 5th and 95th percentiles, respectively. The letters above the boxes represent statistical significance, and regions with no letters in common are considered different from one another (Kruskal-Wallis with Dunn pairwise post hoc test, *p* < 0.05).

**Fig. 3.**
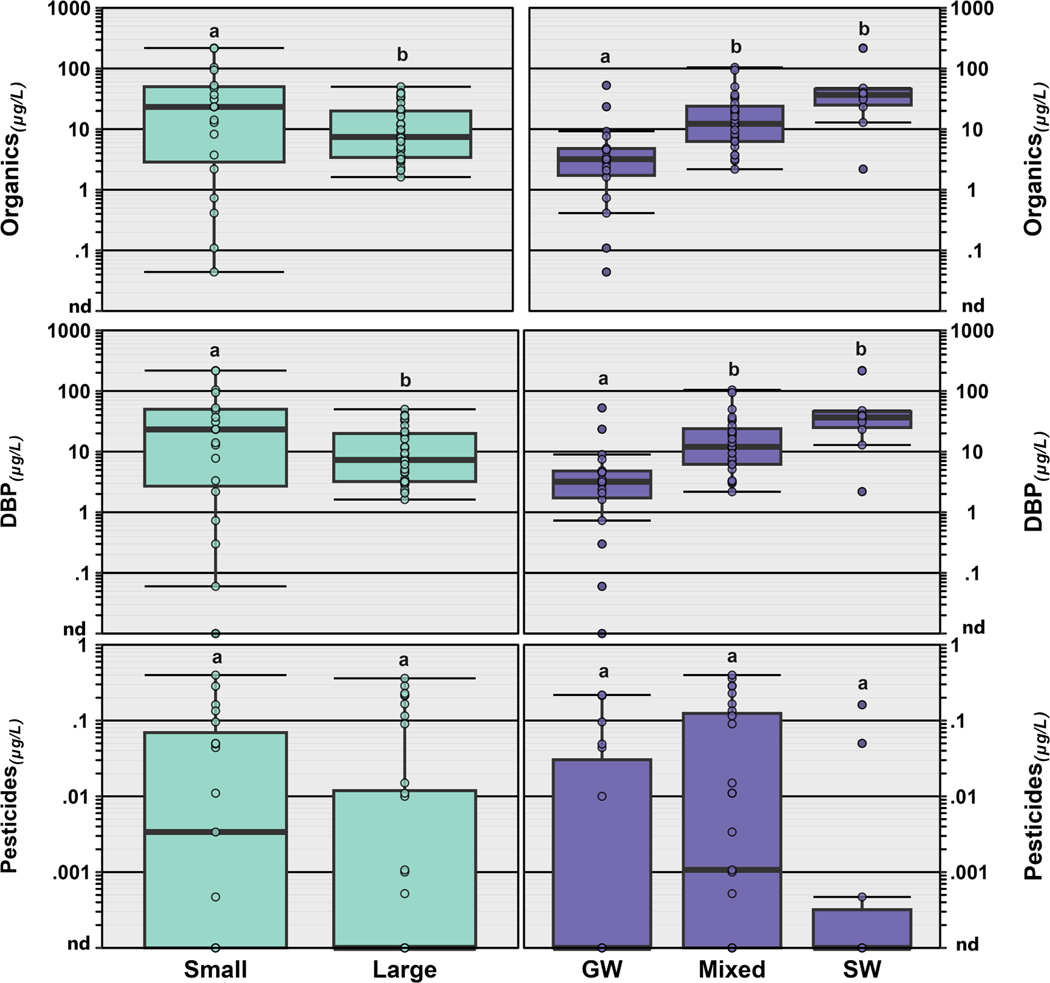
Differences in concentrations (μg/L) of cumulative organics (top), disinfection byproducts (DBP; middle) and pesticides (bottom) in tapwater based on the size of the public water supply (PWS) serving each household (**Left**) and the PWS water source (groundwater (GW), mixed or surface water (SW); **Right**). Small public water supply (PWS) serves <10,000 people while a large PWS serves greater than 10,000 people. A mixed source includes PWS, with both groundwater and surface water sources. In both plots, open circles are data for individual samples. Boxes, centerlines, and whiskers indicate interquartile range, median, and 5th and 95th percentiles, respectively. The letters above the boxes represent statistical significance, and boxes with no letters in common are considered different from one another (Kruskal Wallis with Dunn pairwise post hoc test, *p* < 0.05). Information on PWS size and source water was obtained from the California Safe Drinking Water Information System (SDWIS; https://sdwis.waterboards.ca.gov/PDWW/index.jsp ).

Multiple detections of both regulated and unregulated contaminants were observed in tapwater samples collected one time each within five California regions (Figs. 1–3; Table 1; Figure S1; Tables S3-S4). Concentrations in our samples were compared by region, drinking-water source (groundwater, surface water, mixed), and PWS service population (small [<10,000 served], large [>10,000 served]). To assess potential human-health concerns in tapwater collected throughout our study region, contaminant concentrations were also compared to existing regulatory standards (MCL) and available health-based benchmarks (Table 1, Table S10). It is also important to note that based on the results of the brief survey administered, 30 % of participants reported drinking unfiltered tapwater and an additional 32 % reported using it for cooking or making hot beverages, while 19 % of the participants used unfiltered tapwater only for other activities, and 5 % of participants (3 out of 60) reported that they did not use unfiltered tapwater at all. We do not have information on whether residences had home point-of-use-filters or relied on bottled water as their sole drinking water source.

### 3.1. Occurrence of contaminants in California tapwater

Of the 251 unique organic compounds that were analyzed in this study, 54 (22 %) were detected at least once (Figure S1; Table S3), with detections per sample ranging from 0 to 23 (median: 5). DBPs were observed most frequently (90 %) followed by pesticides (42 %), PFAS (30 %) and pharmaceuticals (23 %). Thirteen DBPs were detected, including bromodichloromethane (87 % of samples), chloroform (85 % of samples), dibromochloromethane (75 % of samples), bromoform (72 % of samples) (Table S3; Figure S1) as well as several iodinated haloacetonitriles and halonitromethanes. Three residences in our study, served by small groundwater sourced PWS (Kern: 2; Merced/Fresno: 1) had either no DBPs detected (site 030) or low concentrations of either bromoform (0.06 μg/L; site 031) or chloroform (0.3 μg/L; site 046), consistent with limited to no disinfection treatment. As expected, and similar to other studies (Bradley et al., 2018, 2023), no DBPs were observed in any private well all of which were located in Gold Country. Detected individual DBP concentrations across our study area ranged from 0.04 to 70.7 μg/L (median: 1.09 μg/L). DBP concentrations made up > 80 % of the total organic concentration in 52 of 55 PWS locations, with no differences in profiles and concentrations by region (Fig. 2). However, DBP concentrations were higher in residences served by small PWS (*p* = 0.0024) and PWS sourced from a surface water (*p* = 0.0001), or mixed sources (*p* = 0.0052) compared to PWS sourced from groundwater (Fig. 3).

Of the 21 pesticides and pesticide transformation products observed, atrazine (28 % of samples) and simazine (22 % of samples) were most frequently detected (Table S3), with detected concentrations ranging from 0.001 to 0.165 μg/L (median: 0.030 μg/L) and 0.005–0.340 μg/L (median: 0.058 μg/L), respectively (Table 1). Some regional differences in the types and concentrations of pesticides detected were observed (Fig. 2) with no differences by PWS size or source water (Fig. 3). Concentrations of pesticides were higher in SELA compared to Kern (*p* = 0.0300) and Gold Country (*p* = 0.0011) with no other regional differences (Fig. 2).

Seven of the 32 analyzed PFAS were detected at least once across all samples. At least one PFAS was detected in 30 % of the homes (18/60); 16 of those were homes in SELA (Table S3). Of the homes where PFAS was detected, 55 % (10/18) had more than one PFAS and one home had six individual PFAS detected (Table S3). One PFAS was detected in a sample collected from a private well in Gold Country (PFOS), and three were observed in one residence from Merced/Fresno counties (PFHxS, PFPeA and PFOA). PFBA was detected most frequently, in 20 % of the samples (all from SELA), followed by PFOA and PFOS, each in 16 % of the samples (Figure S2; Table S3). Individual concentrations of detected PFAS ranged from 0.002 to 0.024 μg/L (median: 0.008 μg/L), with total PFAS concentrations (sum of all PFAS detected) ranging from 0.003 to 0.051 μg/L (median: 0.018 μg/L). Because PFAS was detected primarily in SELA, we were unable to compare across region, PWS size or source water.

Of the 113 human-use pharmaceuticals analyzed, thirteen were detected at least once, with carbamazepine the most frequently detected (12 % of samples) and only in SELA (*n* = 5) and Merced/Fresno counties (*n* = 2) (Table S3; Fig. 2). Only one pharmaceutical (gabapentin) was detected in a private well (Gold Country site 007); no other pharmaceuticals were found in any other samples sourced solely from groundwater (Table S3). Concentrations of detected pharmaceuticals ranged from 0.001 to 0.374 μg/L (median: 0.01 μg/L) with no differences among regions (Fig. 2), PWS size or source water type.

Lastly, despite the detection of potential endocrine-active compounds in tapwater, all bioassay results were negative for AR, GR, TR and AhR (Figures S3-S7). A single sample collected from a private well in Gold Country (site 011; ID-03,131) was marginally positive for ER (1.0 ng estradiol (E2) equivalents/L; Figure S6) but did not exceed the effects-based trigger value (3.8 ng E2-equivalents/L) (Brand et al., 2013), which is indicative of a potential adverse health outcome.

**Fig. 4.**
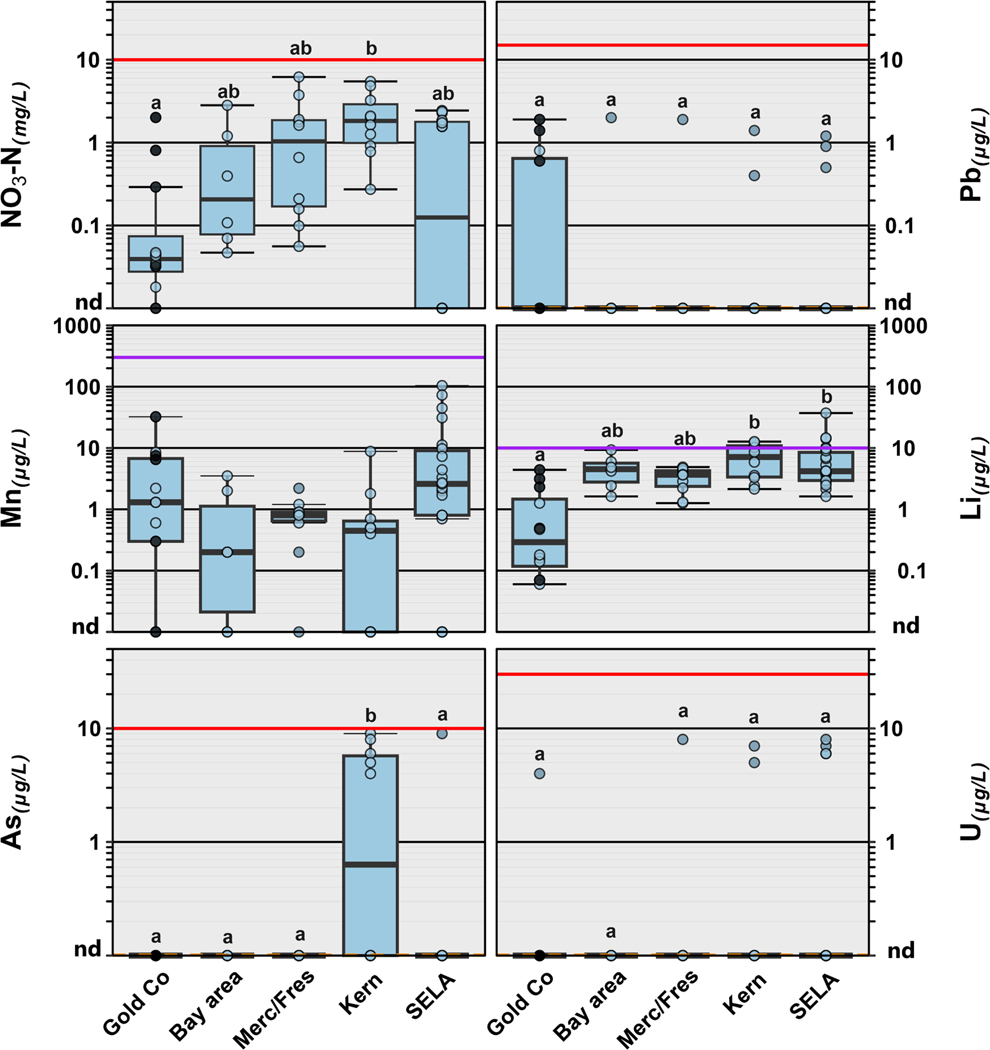
Concentrations (mg/L) of nitrate-nitrogen (NO_3_–N; top left) and concentrations (μg/L) of lead (Pb; top right), manganese (Mn; middle left ), lithium (Li; middle right), arsenic (As; bottom left) and uranium (U; bottom right) in tapwater samples collected in 2020–21 from five regions of California including Gold Country (Gold Co), the San Francisco Bay Area (Bay area), Merced and Fresno counties (Merc/Fres), Kern County (Kern) and southeast Los Angeles (SELA). Private well samples collected from Gold Country are represented by closed circles. Open circles are data for individual public supply samples. Boxes, centerlines, and whiskers indicate interquartile range, median, and 5th and 95th percentiles, respectively. For each element, red colored lines indicate health-based National Primary Drinking Water Regulation Maximum Contaminant Level (MCL: NO_3_–N, As, U) or non-health-based National Primary Drinking Water Regulation Action Level (Pb) and purple lines represent non-enforceable EPA Drinking Water Health Advisory (Mn) or Health-Based Screening Levels (HBSL; Li). The MCL Goals (MCLGs) represented as the orange line on the x-axis for As, Pb and U are zero. The letters above the boxes represent statistical significance, and regions with no letters in common are considered different from one another (Kruskal Wallis with Dunn pairwise post hoc test, *p* < 0.05).

**Fig. 5.**
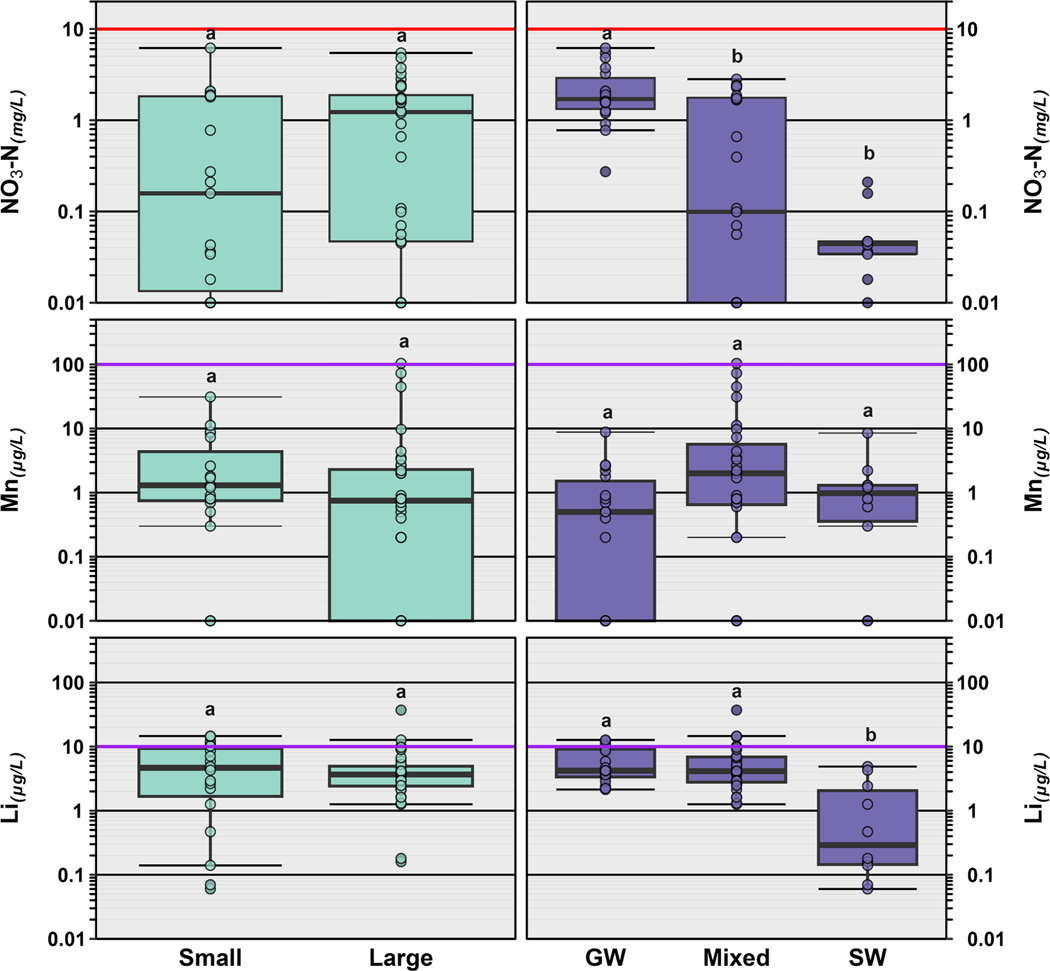
Differences in concentrations of nitrate-nitrogen (NO_3_–N in mg/L; top), manganese (Mn in μg/L; middle) and lithium (Li in μg/L; bottom) in tapwater based on the size of the public water supply (PWS) serving each household (**Left**) and the PWS water source (groundwater, mixed or surface water; **Right**). Small public water supply (PWS) serves <10,000 people while a large PWS serves greater than 10,000 people. A mixed source includes PWS, with both groundwater and surface water sources. In both plots, open circles are data for individual samples. Boxes, centerlines, and whiskers indicate interquartile range, median, and 5th and 95th percentiles, respectively. Red colored lines indicate health-based National Primary Drinking Water Regulation Maximum Contaminant Level (MCL, NO_3_–N) and purple lines represent non-enforceable EPA Drinking Water Health Advisory (Mn) or Health-Based Screening Levels (HBSL; Li). The letters above the boxes represent statistical significance, and boxes with no letters in common are considered different from one another (Kruskal Wallis with Dunn pairwise post hoc test, *p* < 0.05). Information on PWS size and source water was obtained from the California Safe Drinking Water Information System (SDWIS; https://sdwis.waterboards.ca.gov/PDWW/index.jsp).

Twenty-eight of the 32 (88 %) inorganic constituents were detected at least once across all 5 study regions (Table S4). Herein, we specifically focus on six inorganic constituents that are of potential human health concern, including arsenic, uranium, lithium, lead, manganese, and nitrate (Table 1; Fig. 4) at concentrations measured in tapwater. Arsenic was observed infrequently (10 % of samples), with the most detections/highest concentrations occurring in Kern County (Fig. 4; Table S4) and concentrations ranging from 4 to 9 μg/L. Similarly, uranium was observed in 13 % (8 of 60 samples), with concentrations ranging from 4 to 8 μg/L (Fig. 3; Table S4). Manganese was detected in 78 % of the samples (47/60), with concentrations ranging from 0.20 to 104 μg/L (median: 1.80 μg/L; Fig. 3, Table S4) and no differences in concentration based on PWS size or water source (Fig. 5). Lithium was observed in 100 % of samples collected, with concentrations ranging from 0.06 to 37.1 μg/L (median: 3.67 μg/L; Fig. 3, Table S4). Residences receiving water from PWS sourced solely from surface water tended to have lower lithium concentrations, compared to those from either groundwater (*p* = 0.002) or mixed sources (*p* = 0.0046; Fig. 5). Lead was detected in 11 tapwater samples, including four in Gold Country (3 private wells, 1 PWS), one in the Bay Area, two in Kern County, one in Merced/Fresno counties, and three in SELA (Fig. 4; Table S4). Detected concentrations across our study area were low (median: 1.2 μg/L, range 0.5–2.0 μg/L); drinking-water lead contamination is generally attributed to legacy use in service lines and premise plumbing (Levallois et al., 2018; Navas-Acien et al., 2007; Triantafyllidou et al., 2021). Lastly, nitrate as nitrogen was observed in 78 % of the samples collected, with concentrations ranging from 0.018 to 6.19 μg/L (median: 1.56 μg/L; Table 1; Table S4) and higher concentrations observed in the agricultural Central Valley (Fig. 4). Median concentration of nitrate was also higher in tapwater sourced from groundwater compared to surface water (*p* = 0.0006) or mixed sources (*p* = 0.0027), with no differences based on the size of the PWS (Fig. 5).

### 3.2. Comparison to federal drinking water regulations

Federal regulatory standards are available for 20 of the constituents observed in our study area including 11 organics (4 DBPs, 2 pesticides, 4 PFAS) and 9 inorganics (Table 1). Over 90 % of the samples collected were from residences on PWS, and we observed very few MCL exceedances apart from total trihalomethanes (TTHM) and several PFAS. Three residences, two in Merced/Fresno (sites 042, 043) and one in SELA (site 056) had TTHM concentrations that exceeded the regulatory standard of 80 μg/L (Table 1; Table S3). The two residences in Merced/Fresno were served by the same small PWS (population served: 462) receiving water from a surface water source, while the SELA residence was served by a small PWS (population served: 9500) receiving water from a mixed surface-water and groundwater source. On April 10, 2024 EPA released the final rule for PFAS, setting regulatory standards (Table 1; Table S3) for five individual PFAS (PFOA, PFOS, PFNA, PFHxS and GenX; U.S. Environmental Protection Agency, 2024b). The MCL for PFOA and PFOS (4 ng/L) were exceeded in 15 % and 17 % of the residences, respectively, with all but one (a private well in Gold Country) exceedance occurring in residences from SELA (Table S3; Von Behren et al., 2024); no other PFAS MCL exceedance was observed (Table 1). EPA also established a Hazard Index Level (HI=1) for two or more of four PFAS (PFNA, PFBS, PFHxS and GenX) as a mixture (U.S. Environmental Protection Agency, 2024b). It is important to note, that the newly established MCL for PFOA, PFOS, PFNA, PFHxS and GenX were at or below the analytical reporting limits used during this study (Table S2); so, values between the MCL and reporting limits are considered underreported. No tapwater samples exceeded the HI of 1 for the EPA designated PFAS mixture.

However, to inform risk to vulnerable subpopulations (e.g., infants, children, pregnant women, elderly and immunocompromised), we also compared our data to MCLG (Table 1; Table S3), which typically provide an exposure concentration below which there is no known risk of an effect, with an adequate margin of safety (U.S. Environmental Protection Agency, 2024a). We observed multiple MCLG exceedances for several DBPs (Table 1) including *de facto* exceedances of MCLG of zero for all residences with detectable levels of both bromodichloromethane (87 %) and bromoform (72 %) as well as exceedances of dibromochloromethane (MCLG: 60 ug/L) in 3 % of the residences (Table 1; Table S3). Five of the detected inorganics have established MCLG (arsenic, copper, lead, nitrate, and uranium; Table 1). The MCLG (zero) for arsenic, lead, and uranium were exceeded (i.e., detected) in, 10 %, 18 % and 13 % of the samples, respectively (Table S4; Fig. 4).

### 3.3. Tapwater effects-based screening assessments

**Fig. 6.**
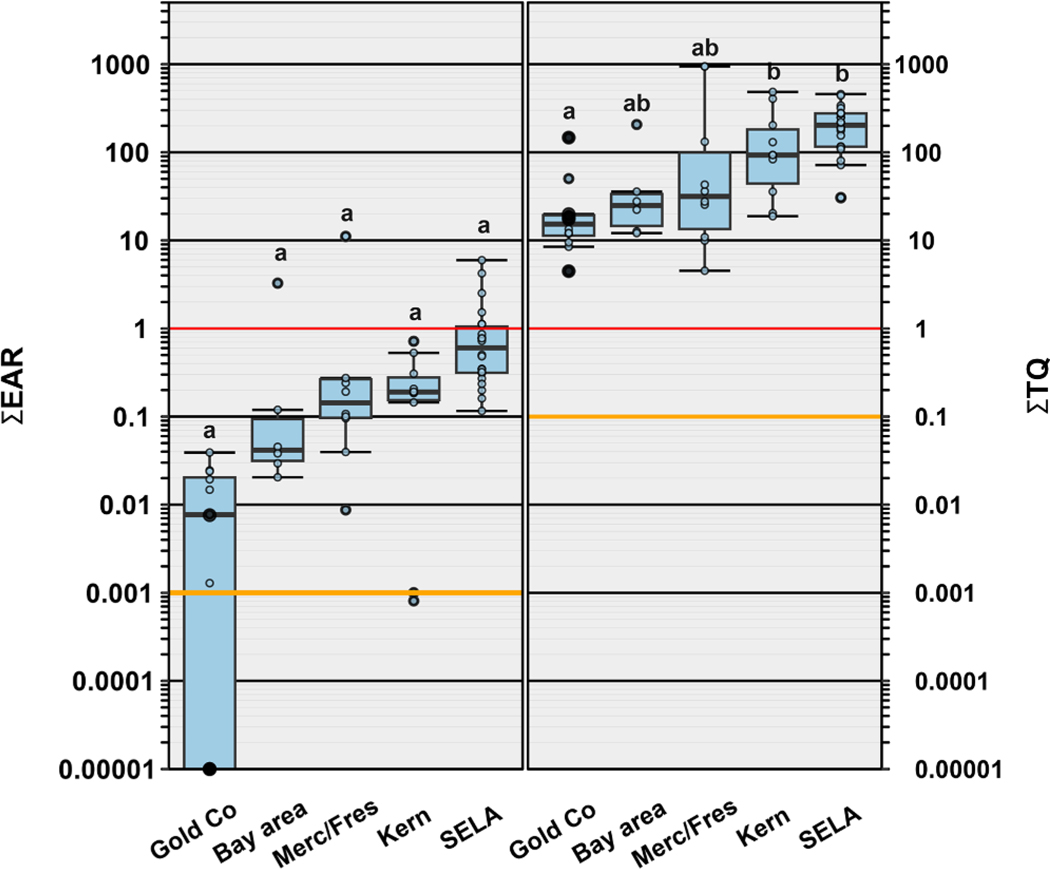
**Left.** Cumulative maximum exposure-activity ratios (ΣEAR) across all assays for 33 analytes listed in ToxCast and detected in tapwater samples. Solid red and yellow lines indicate concentrations shown to modulate effects in vitro and effects-screening-level thresholds (EAR = 1 and EAR = 0.001), respectively. **Right.** Human health benchmark cumulative toxicity quotient ($\left(\sum \text{TQ}\right)$) for inorganic and organic analytes listed in Table S10 and detected in tapwater samples. Solid red and yellow lines indicate benchmark equivalent concentrations and effects-screening-level threshold of concern (TQ = 1 and TQ = 0.1), respectively. In both plots, tapwater samples were collected in 2020 from five regions of California including Gold Country (Gold Co), the San Francisco Bay Area (Bay area), Merced and Fresno counties (Merc/Fres), Kern County (Kern) and southeast Los Angeles (SELA). Private well samples collected on from Gold Country are represented by closed circles. Open circles are data for individual public supply samples. Boxes, centerlines, and whiskers indicate interquartile range, median, and 5th and 95th percentiles, respectively. The letters above the boxes represent statistical significance, and boxes with no letters in common are considered different from one another (Kruskal Wallis with Dunn pairwise post hoc test, *p* < 0.05).

Of the 54 organics detected, 37 had exact Chemical Abstract Services number matches in the ToxCast database but only 33 had an EAR>0.00001 (Tables S8–9). ∑EAR ranged from <0.00001 to 11.1 (median: 0.203; IQR: 0.039–0.719). Although we observed no differences among regions (Fig. 6), the lowest ∑EAR was observed in samples collected from Gold Country (median: 0.0077), followed by those from the Bay Area (median:0.042), the Central Valley (Merced/Fresno (median: 0.149), and Kern Counties (median: 0.190)), with the highest values observed in tapwater from SELA (median: 0.614). A ΣEAR>1 (solid red line in Fig. 6) indicates cumulative exposure concentrations capable of modulating molecular level effects in vitro and a ∑EAR=0.001 is employed as a precautionary screening level (yellow line, Fig. 6) (Bradley et al., 2018), with reported approximate equivalence to ∑TQ=0.1 (Corsi et al. 2019). Nine tapwater samples across three of the five regions including one in the Bay Area, two in Merced/Fresno Counties and six in SELA had a ΣEAR>1 (Fig. 6; Table S8), all attributable to the DBP dibromochloromethane (Figure S8; Table S8). Fifty-four tapwater samples exceeded the ∑EAR>0.001 driven primarily by dibromochloromethane and other DBP and to a lesser extent the pharmaceutical fluticasone propionate (a corticosteroid used to treat skin conditions such as eczema and psoriasis) and various pesticides (Figure S8, Table S8). Lastly, four of the five (80 %) of private well samples (Gold Country only) did not exceed a ∑EAR=0.001 precautionary screening level (Fig. 6; Table S8), consistent with lack of disinfection treatment and thus DBP detections.

We also used a benchmark based ΣTQ approach to estimate the cumulative human-health risk of exposure to both organic and inorganic contaminants in tapwater samples collected throughout California. Human health benchmarks were available for 48 of the 82 (58 %) detected organic and inorganic contaminants in our study. All tapwater samples collected across all regions exceeded a ΣTQ of 1 (median 18.8; IQR: 19.7–209; Fig. 6), indicating a high probability of aggregated risk. Median ΣTQ were higher in SELA (*p* < 0.0001) and Kern County (*p* = 0.043) compared to Gold Country with no other regional differences noted (Fig. 6). However, the types of contaminants driving the exposure risk varied by region and by infrastructure-type (private well, PWS) (Table S10). The number of individual contaminants exceeding a ΣTQ of 1 ranged from 1 to 6 (median: 2.5) and were driven predominately by bromodichloromethane and tribromomethane in PWS tapwater samples. ΣTQ exceedances ranged from 1 to 3 in taps supplied by PWS in Gold Country, the Bay Area and Merced/Fresno Counties driven by DBPs (bromodichloromethane, tribromomethane, dibromochloromethane) and to a lesser extent PFOS, PFOA, arsenic, uranium, and lead (Table S11; Figure S9). However, in Kern County exceedances ranged from 2 to 6 and in SELA exceedances ranged from 2 to 4 most frequently driven by DBP, PFOA and PFOS (SELA only) as well as arsenic, uranium, lead, and lithium (Table S11; Figure S9). Exposure risks in private-well tapwater samples collected only from Gold Country were dominated by lead and, in one sample, cadmium and nitrate (Table S11).

## 4. Discussion

Drinking water is a route of human exposure to a variety of natural and anthropogenic contaminants, globally. Recently, public concerns over drinking water quality have become apparent as more studies begin to address human-health effects to low-level contaminant mixture exposures (Carlin et al., 2013; Cui et al., 2016). Because, this perception of both relative safety and acceptable risk vary widely across individuals, communities and cultures (Hrudey, 2009) assessments that reflect both environmental contaminant complexity and human-health implications are needed to support and encourage community engagement and to inform decision making (Bradley et al., 2021a). The current study was designed as an initial reconnaissance (one-time, synoptic approach) to provide information on exposure to and potential human-health effects of contaminant mixtures in drinking water at the point of consumption from a select number of communities identified as disadvantaged in five regions of California. The study was not an exhaustive assessment of the state’s drinking water quality, was designed as an initial assessment in communities often overlooked during routine monitoring and does not account for external factors affecting drinking water quality (e.g., seasonality). Further, because all communities selected were considered disadvantaged by meeting two of the three established criteria, the information and comparisons presented may not be reflective of all PWS and private wells in California. However, the data provided can help communities to identify potential drinking water concerns and to seek support for further monitoring and improved drinking-water treatment, as warranted.

Similar to results from other studies at the tap (Bradley et al., 2020, 2018, 2021a; Bradley et al., 2021b, 2022; Smalling et al., 2023a), we observed frequent detections of both regulated and unregulated contaminants in tapwater samples throughout our five California regions (Figs. 1–2,4; Table 1; Figure S1; Tables S3-S4) with limited bioassay response, similar to others (Conley et al., 2017; Medlock Kakaley et al., 2020, 2021) and no exceedances of the effects-based trigger value (Brand et al., 2013). This is consistent with that fact that most of the analytes were observed at low μg/L concentrations, are not currently regulated under the Safe Drinking Water Act, and thus not targets for compliance monitoring in PWS. Co-occurring detections of contaminants that are known to undergo significant changes within the PWS distribution system (e.g., DBP) and the premise plumbing (e.g., lead, copper) emphasize the importance of monitoring for a robust mixture of contaminants at the tap that more realistically reflect documented environmental complexities (e.g., source water) (Bradley et al., 2017) and acknowledge potential changes during the treatment process and distribution.

For those samples served by PWS, the types of contaminants detected, and concentrations varied by region, drinking water source, and PWS service population (small [<10,000 served], large [>10,000 served]; Fig. 2–5), indicating the continued need for these types of broad assessments to accurately characterize potential human-health concerns for communities reliant on a variety of water sources. Regionally, arsenic concentrations were highest in Kern County with limited detections in other regions, while nitrate and lithium were lowest in Gold Country compared to other regions (Fig. 4; Table S4). Elevated detections of arsenic in Kern County could be due in part to past agricultural uses in pesticides or animal antibiotics (Punshon et al., 2017). Communities in SELA, on the other hand, were exposed to greater numbers of contaminants, including PFAS, and to higher concentrations of pesticides, compared to the other regions we sampled (Fig. 2). Unlike other parts of southern California that import their water from northern California, extensive aquifer storage exists under much of the LA metropolitan area (Amter and Ross, 2001; Reibel et al., 2021), and much of SELA is reliant on groundwater recharge which can be a source of contaminants like PFAS to aquifers (Edwards et al., 2022; Reibel et al., 2021). However, channelization of rivers, paving of flood plains, and increasing water use have diminished groundwater recharge and depleted groundwater resources, prompting engineered groundwater-recharge efforts (Reibel et al., 2021). Because many LA-area PWS are dependent partially on groundwater wells (Amter and Ross, 2001; Reibel et al., 2021), these recharge efforts (extensive infiltration galleries) combined with rapid industrial development of the area have degraded drinking-water quality.

Further, differences in the type of drinking water source (surface water, groundwater, mixed) likely drive the types of contaminants present in household tapwater. Groundwater-only sources tended to have higher concentrations of inorganics, whereas surface-water-only sources had greater numbers of organics. Consistent with the well-documented occurrence of elevated groundwater nitrate concentrations in California (Burow et al., 2008; Rosenstock et al., 2014), higher nitrate concentrations were observed in taps sourced from groundwater compared to those from surface-water or mixed sources (Fig. 5). Similarly, lithium concentrations in groundwater-sourced samples tended to be higher than those reported in surface-water-sourced samples, consistent with the elevated concentrations of dissolved solids observed in groundwater and documented importance of groundwater as a source of lithium to surface water (Sharma et al., 2022). Mixed-source samples, 74 % of which were in SELA, had a greater number of compounds detected, compared to surface-water and groundwater only samples. Cumulative concentrations of DBP (>80 % of total) and other organics were lower in groundwater-sourced samples than in surface-water- or mixed-source samples, as expected (Fig. 3). DBP formation is a result of organic matter in the drinking-water source and groundwater typically has low organic-matter concentrations compared to surface water (Alexandrou et al., 2018; Li and Mitch, 2018). Although, no differences in inorganics by PWS size were observed, potentially due to relatively small sample sizes, elevated drinking-water-nitrate exposures have been reported previously for communities depending on small, groundwater-sourced PWS (Bangia et al., 2020; Schaider et al., 2019). Bangia et al. (2020) observed a positive trend in TTHM concentration with system size in California and hypothesized that chlorine-disinfection levels and resulting DBP exposures are greater for large systems. In contrast, in this study, cumulative concentration of DBP and other organics were higher in residences served by small PWS compared to those served by large PWS (Fig. 3), with no systematic difference in the number of detected organic contaminants with PWS size. For pesticides and pharmaceuticals, we observed no difference by PWS size or drinking-water source, potentially due to the high number of non-detects and relatively small sample sizes (Fig. 3). Small PWS tend to have limited resources compared to their larger counterparts, struggle to meet regulations, and often rely heavily on chlorine disinfection due to lower microbial-quality source waters (U.S. Environmental Protection Agency, 2011b).

We also observed exceedances of MCL (e.g., PFOA, PFOS and TTHM) and MCLG (e.g., arsenic, lead, uranium, DBP, and PFAS) for several constituents primarily in public supply tapwater (Table 1; Tables S3 and S4). For those receiving water from a PWS, fourteen samples exceeded at least one MCL while half of those exceeded two MCLs. MCL violations in CA are common, particularly in small PWS that serve rural low-income communities with fewer than 500 service connections (State Water Resources Control Board, 2022). Public-supply MCL were exceeded in about 25 % of our samples of which half were from residences served by small PWS. Similar to other studies by this group (Bradley et al., 2020; Smalling et al., 2023a), all 55 residences served by a PWS exceeded at least one MCLG, 19 had at least two exceedances and 10 residences had 3 to 5 MCLG exceedances (Tables S3, S4). Although, multiple exceedances of MCL-equivalent concentrations of inorganics (e.g., arsenic, lead and uranium) and PFAS have been reported in private wells in other tapwater studies (Bradley et al., 2021a, 2022; Smalling et al., 2023b), no MCL exceedances and only 4 MCLG exceedances (lead: 3 samples; PFOS: 1 sample) were observed in the limited number of private-well samples in this study (5 samples in Gold Country).

Exposures to lead through drinking-water are of particular human-health concerns to vulnerable subpopulations including formula-fed infants, children, pregnant women and breast-feeding mothers and can result in fetal death, reduced birth rates, neurocognitive impairment, as well as cardiovascular disease and related mortality (Lanphear et al., 2016, 2018; Levallois et al., 2018; Navas-Acien et al., 2007). Drinking-water lead contamination is generally attributed to legacy use in service lines and premise plumbing (Levallois et al., 2018; Navas-Acien et al., 2007; Triantafyllidou et al., 2021) and median concentrations were about 1 μg/L similar to national mean lead levels (Bradham et al., 2023) but lower than those reported from areas with documented lead issues (Roy and Edwards, 2019). However, it is important to note that lead concentrations reported herein likely do not represent a worst-case scenario because flushing of service lines or premise plumbing can reduce lead concentrations (Triantafyllidou et al., 2013) and same day prior use was typical. Further, if more affluent members of our communities volunteered as noted by others (Flanagan et al., 2015; Wait et al., 2020), then premise plumbing may be better maintained than for the community at large thus associated exposures could be biased low.

On average California has some of the greatest numbers of systems violations and populations served with systems violations for nitrate (Pennino et al., 2017). Although, nitrate was observed frequently particularly in the Central Valley (Merced/Fresno and Kern counties), concentrations were well below the MCL and MCLG of 10 mg/L (median: 1.56 mg/L; Table 1) established to protect bottle fed infants against methemoglobinemia. However, more recently nitrate exposure has been linked to other adverse health outcomes including some cancers (Jones et al., 2017), thyroid disease (Aschebrook-Kilfoy et al., 2012) and neural tube defects (Brender et al., 2013) at concentrations closer to 1 mg/L.

Arsenic and uranium occur naturally in the environment and both have been detected in groundwater wells throughout California at mean concentrations (arsenic: >1–3 μg/L and uranium: >1– 8 μg/L; Bangia et al., 2020) depending on region similar to what were observed in this study (Table 1). Frequent detections in drinking water resources throughout California places a heavy burden on smaller PWS who struggle to comply with existing regulations (Bangia et al., 2020; Martinez-Morata et al., 2022; Thiros et al., 2015). Although arsenic and uranium were observed infrequently in our study, human-health risks below their respective MCLs have been documented previously. Exposure to arsenic through drinking water has been associated with increased risk of several cancers, cardiovascular disease, diabetes, (Mohammed Abdul et al., 2015; Navas-Acien et al., 2005, 2008), adverse pregnancy outcomes and mortality (Argos et al., 2010; Shih et al., 2017). Uranium exposure has been linked with nephrotoxicity and osteotoxicity in humans (Kurttio et al., 2005; Magdo et al., 2007).

Manganese is considered an emerging contaminant of concern due to its potential cognitive and behavioral effects to children (Juchnowicz et al., 2020; Rahman et al., 2017; Schullehner et al., 2020) at concentrations below EPA’s lifetime health advisory of 300 μg/L (U.S. Environmental Protection Agency, 2004). In our study, manganese was detected frequently (median: 1.6 μg/L) with no exceedances of the EPA health advisory (300 μg/L) or the WHO provisional value (80 μg/L; Fig. 3). A recent study reported mean manganese concentrations of about 10 μg/L in over 2800 California PWS that report manganese concentrations at the point-of-entry, and exceedances of the WHO provisional value were primarily observed in small PWS with little to no exceedances in large PWS (Aiken et al., 2023).

Lithium, is observed frequently in drinking water, is not currently regulated in the U.S, is used to treat depression and bipolar disorder (Curran and Ravindran, 2014), and low-level exposure through drinking water has been linked to positive mental health benefits (Eyre-Watt et al., 2021; Knudsen et al., 2017). For example, it has been hypothesized that naturally occurring lithium in drinking water has the potential to reduce the risk of suicide and act as a mood stabilizer particularly in populations with high suicide risks (Memon et al., 2020). More recently, however, a link between elevated drinking-water lithium exposures and autism and disruption of thyroid hormone levels has been suggested (Broberg et al., 2011; Liew et al., 2023). Based on the EPA’s provisional reference dose of 2 μg/kg body weight per day (Norman et al., 2018), the USGS developed a non-enforceable HBSL of 10 μg/L, which was exceeded in 13 % of our samples (8/60) (Fig. 4; Table S4). Although concentrations fell mostly below the HBSL, median concentrations (3.7 μg/L) in our study were similar to those reported previously in surface water sources (median: 3.9 μg/L; (Sharma et al., 2022)) but lower than those reported in groundwater sources (median: 8.1–13.9 μg/L; (Lindsey et al., 2021; Sharma et al., 2022)). Lithium is ubiquitous in drinking water sources across the U.S.; thus, more information is needed at the point of consumption to assess exposure more adequately and to further understand the potential human-health effects.

PFAS advisories and regulations have been changing rapidly over the last few years as more information on exposure and toxicity have become available (Post, 2021). PFAS are persistent in the environment (Evich et al., 2022) and in humans (Hu et al., 2019), are considered a human-health concern (Liu et al., 2018; Sunderland et al., 2019), and have been observed in both drinking water resources (McMahon et al., 2022; Sims et al., 2022) and tapwater (Smalling et al., 2023b). Although, federal regulations for six individual PFAS were promulgated in the U.S. in 2024, the 32 PFAS targeted in this study, of which 7 were detected represent only a fraction of potential PFAS in the environment today (Glüge et al., 2020; Wang et al., 2022). The median cumulative PFAS (sum of all PFAS detected) concentration across our study area was 0.018 μg/L and similar to those reported recently in a study assessing PFAS occurrence and concentrations in tapwater throughout the U.S (Smalling et al., 2023b). The MCL for PFOS and PFOA were exceeded in 10 and 9 of the 10 samples in which they were detected (all *de facto* MCLG ‘zero’ exceedances; Table 1). Although, PFAS were detected relatively infrequently across the entire study area, regulatory exceedances in about half the residences sampled in SELA indicate these communities may be disproportionately affected by PFAS. DBP are another class of well documented drinking water contaminants, and median TTHM concentrations (7.6 μg/L) in our study were within the range (8.9–38.7 μg/L) of what has been reported in PWS throughout California (Bangia et al., 2020). The public health benefits of disinfection as a means of preventing water borne diseases in drinking-water infrastructure in the U.S. and globally are well established (Richardson and Postigo, 2012). However, detection of both regulated and unregulated DBP, in some PWS above a regulatory standard, are public health concerns (Reynolds et al., 2008; Schoenen, 2002).The carcinogenic and genotoxic effects of regulated DBP are well documented (Villanueva et al., 2014), as are the linkages between exposure and blood DBP concentrations (Rivera-Núñez et al., 2012), and the associations with bladder cancer (Hrudey et al., 2015; Hrudey and Fawell, 2015). Further, 69 % of the DBP (9 out of 13) observed in this study, which included iodinated haloacetonitriles and halonitromethanes, are unregulated and rarely monitored but are considered more toxic than regulated THMs and haloacetic acids (Muellner et al., 2007; Richardson and Plewa, 2020; Wagner and Plewa, 2017). The frequent detections of DBP and PFAS in tapwater, some of which exceeded an MCL are consistent with previous studies (Bradley et al., 2020; Smalling et al., 2023b) and support the need for continued assessments of mixtures in tapwater particularly in disadvantaged communities and an improved understanding of cumulative health-risks of DBP and PFAS.

Results illustrate that the communities sampled are exposed to low-level contaminant mixtures (including putative endocrine-disrupting chemicals) with poorly understood human-health implications cumulatively. Pesticides and pharmaceuticals, most of which are not regulated in drinking water, are designed to be bioactive, generally targeting molecular endpoints. Many known DBP and PFAS are considered potential carcinogens and can have negative effects on the immune system (Barry et al., 2013; Grandjean and Budtz-Jørgensen, 2013; Liu et al., 2018; Richardson and Postigo, 2012). Disadvantaged communities are often disproportionately affected by environmental contamination and are considered more vulnerable to adverse health outcomes compared to other populations (Brown, 1995). A recent study found that PWS watersheds serving a greater proportion of people of color as well as residents living at or below the poverty line were more likely to be located near PFAS sources and have detectable levels of PFAS in drinking water (Liddie et al., 2023), supporting the need for continued characterization of drinking-water contaminant-mixture exposures particularly in underserved communities where this information may be lacking. Currently, limited information is available on inequities in drinking water quality as it relates to organic contaminants (Smalling and Bradley, 2024) because these types of studies focused on a large suite of regulated and unregulated contaminants are lacking broadly across the U.S. and globally making it difficult to determine which exposures directly affect human health. To adequately address inequities in drinking water as it relates human health, studies must include a robust assessment of exposure everywhere (Cui et al., 2016) with an emphasis on a range of communities (poor vs wealthy), drinking water types (public vs. private), PWS sizes and drinking water source waters.

Detections of complex mixtures of organic and inorganic contaminants as well as multiple exceedances of health-based benchmarks further supports the need for a cumulative or aggregated assessment of these mixtures in individual taps. To begin to address potential human health effects based on detected contaminant mixtures, two screening approaches (ΣEAR and ΣTQ) were employed. Both approaches assume additivity and do not take into consideration potential synergistic or antagonistic effects of contaminants. The ΣEAR precautionary screening approach utilizes high throughput molecular (in vivo) endpoint data from the ToxCast database to screen over 10,000 unique organics (Blackwell et al., 2017; Bradley et al., 2021a). A limitation to this approach is that in some cases not all predicted molecular responses are adverse at the organismal level and may not accurately reflect apical human health endpoints (Blackwell et al., 2017; Paul Friedman et al., 2019). Because the analytical scope of this study (251 organic analytes) comprised only a fraction of the compounds estimated to be in production globally (Wang et al., 2020), included only a moderate number of environmental degradates (Dobson, 2004), and, notably, did not cover a range of VOC drinking-water contaminants documented previously in California (Williams et al., 2002, 2004), a ΣEAR of 0.001 was deemed appropriate as a precautionary screening level for potential exposure risk to chemical mixtures. Because the ToxCast-based ΣEAR approach does not cover inorganics, we also employed the ΣTQ Hazard Index (HI) approach to assess combined exposure risks for organics and inorganics (Bradley et al., 2021a). Although this approach addresses apical human health endpoints, it is limited by existing health benchmarks.

Results from the precautionary screening assessments indicate that biological activity (EAR) exceeding the precautionary screening level of 0.001 were common and comparable across the communities sampled (Fig. 6). ΣTQ values exceeded an effects screening level of concern (TQ>1) in every tap sampled with notable community differences (Fig. 6). Screening level exceedances were primarily driven by MCLG for DBP, and to a lesser extent PFAS, arsenic, uranium, and lead. These results support the need for a better understanding of exposure-effects relations and cumulative adverse health risks of both anthropogenic and natural contaminants as well as improved treatment to reduce human exposure to these contaminants. Although disinfection is necessary to protect public health, this study also reemphasizes the need for more information on the health effects of both regulated and unregulated DPB (Richardson and Kimura, 2020) as well as for improved treatment pre-disinfection particularly for surface water and mixed sourced PWS to reduce or remove DBP precursors like natural organic matter (Bond et al., 2014). Common exceedances of screening levels of concern were ubiquitous in tapwater collected as part of this study, despite its limited analytical scope compared to other recent more recent USGS studies (Bradley et al., 2018, 2021b, 2022; Smalling et al., 2023a), reinforcing the need to further assess tapwater exposure risk in these and other disadvantaged communities throughout California and nationally.

There are several noteworthy limitations to the study design and approach presented herein. Due in large part to funding constraints and prioritization of population-relevant reconnaissance of a range of exposure points within a given community, this study employed a onetime spatial–synoptic approach, which provided no insight into seasonal variability of source water properties and other factors that could influence drinking water quality. Because our sample size was small and all communities selected were considered disadvantaged, the information and comparisons presented may not be reflective of all PWS and private wells in California. Although households were selected to represent the community’s water supply and not the sociodemographic makeup of the respective community, the inherent participatory bias in which more affluent members of the community are more likely to volunteer for these types of studies (Flanagan et al., 2015; Gibson and Pieper, 2017; Wait et al., 2020) should be considered when interpreting the results. Lastly, assessing human-health risk to low-level contaminant mixture exposures is complicated by the limited number of health benchmarks/regulations and the difficulty is assessing mixture toxicity. The effects screening approaches (ΣEAR and ΣTQ), assumed additive toxicity, do not take into consideration potential synergistic or antagonistic effects of contaminants but are considered an initial first step in addressing potential human-health effects. Despite these limitations, the data provided can help these and other communities identify potential drinking water concerns and seek support for further monitoring and improved drinking-water treatment, as warranted. Future studies could attempt to address many of these limitations by increasing sample size within and between communities, expanding the scope to include both advantaged and disadvantaged communities, increasing the number of private wells sampled, including in depth health and socioeconomic surveys of participants, and establishing a link between water quality sampling an ongoing environmental cohort studies to address actual human health effects.

## 5. Conclusion

Drinking-water quality and quantity in California is complicated by years of drought, expanding populations, over pumping of groundwater, movement of water throughout the state, and groundwater recharge. Currently, there is a paucity of information on low-level exposure to complex mixtures of organic and inorganic contaminants in diverse communities reliant of PWS and private wells. Our study highlights the importance of assessing mixed-contaminant exposures in drinking water to adequately address human-health concerns like breast cancer risk. This study, which utilized a snapshot of disadvantaged communities from five distinctly different regions, is a first step in identifying potential tapwater contaminants of concern and providing a foundation for future studies across a greater number of communities in California. Regulated and unregulated contaminants were prevalent in tapwater collected as part of this study no matter the PWS size, with multiple exceedances of human-health benchmarks indicating the importance of systematic testing of drinking water at the point of consumption (tapwater) with an emphasis in disadvantaged communities in both rural and urban areas where data may be limited. To begin to break down socioeconomic barriers related to drinking water quality, future studies could focus efforts on understanding exposures to contaminant mixtures across a range of PWS, regions, seasons and source water types in California, the U.S. and globally which will provide diverse communities with information to adequately assess their risks and more effectively advocate for improvements in treatment technologies and water quality monitoring.

## Supplementary Material

Supplement

Supplementary material associated with this article can be found, in the online version, at doi:10.1016/j.watres.2024.122485.

## Figures and Tables

**Table 1:** Percent detection frequency, median concentration, human-health-only Maximum Contaminant Level Goal (MCLG) risk-screening benchmark, and public-supply-enforceable Maximum Contaminant Level (MCL) for the 21 (40 % of total) detected constituents with National Primary Drinking Water Regulation standards (see Tables S3-S4, S10 for complete list of detected chemicals and health-screening benchmarks). Numbers in parentheses indicate the range of detected concentrations and number of exceedances of MCLG and MCL, respectively.

Constituent	Class	Detection Frequency	Median (range)	MCLG (# exceedances)	MCL (# exceedances)

Bromodichloromethane	DBP	86.7 %	1.07 μg/L (ND-70.7)	Zero **(52)**	80 μg/L (0)
Bromoform	DBP	71.7 %	2.50 μg/L (ND-47.7)	Zero (43)	80 μg/L (0)
Chloroform	DBP	85.0 %	0.78 μg/L (ND-50.6)	70 μg/L (0)	80 μg/L (0)
Dibromochloromethane	DBP	75.0 %	1.55 μg/L (ND-65.8)	60 μg/L (2)	80 μg/L (0)
Total Trihalomethanes	DBP	90 %	7.6 μg/L (ND-206)	Zero **(54)**	80 μg/L (3)
2,4-D	Pesticide	1.7 %	0.050 μg/L	70 μg/L (0)	70 μg/L (0)
Atrazine	Pesticide	28.3 %	0.030 μg/L (ND-0.165)	3 μg/L (0)	3 μg/L (0)
Simazine	Pesticide	21.7 %	0.058 μg/L (ND-0.340)	4 μg/L (0)	4 μg/L (0)
PFHxS	PFAS	5.0 %	5.0 ng/L (ND-5.0)	10 ng/L (0)	10 ng/L (0)
PFNA	PFAS	3.3 %	2.0 ng/L (ND-2.0)	10 ng/L (0)	10 ng/L (0)
PFOA	PFAS	16.7 %	8.0 ng/L (ND-14.0)	Zero (10)	4 ng/L (9)
PFOS	PFAS	16.7 %	8.0 ng/L (ND-18.0)	Zero (10)	4 ng/L (10)
Arsenic	Trace element	10.0 %	7.0 μg/L (ND-9.0)	Zero (6)	10 μg/L (0)
Barium	Trace element	100 %	68.5 μg/L (0.110–323)	2000 μg/L (0)	2000 μg/L (0)
Cadmium	Trace element	5.0 %	2.0 μg/L (ND-3.0)	5 μg/L (0)	5 μg/L (0)
Chromium	Trace element	58.3 %	2.0 μg/L (ND-11.0)	100 μg/L (0)	100 μg/L (0)
Copper	Trace element	95.0 %	15.3 μg/L (ND-287)	1300 μg/L (0)	1300 μg/L (0)
Lead	Trace element	18.3 %	1.2 μg/L (ND-2.0)	Zero (11)	15 μg/L (0)
Uranium	Trace element	13.3 %	6.5 μg/L (ND-8.0)	Zero (8)	30 μg/L (0)
Fluoride	Major element	85.0 %	0.290 mg/L (ND-0.760)	4 mg/L (0)	4 mg/L (0)
Nitrate-N	Major element	78.3 %	1.56 mg/L (0.018–6.19)	10 mg/L (0)	10 mg/L (0)

[DBP, disinfection byproduct; ND, not detected; PFAS, per-polyfluoroalkyl substances].

## Data Availability

Data in this article are summarized in the Supporting Information Tables S1-S11 and Figures S1-S9 and are publicly available at https://doi.org/10.5066/P9X3XLK3 (Romanok et al., 2021).
